# Time estimations by network of beta globin gene cluster haplotypes linked with Hb D‐Los Angeles [β121 (GH4) Glu → Gln GAA → CAA] mutation in the world populations

**DOI:** 10.1002/mgg3.499

**Published:** 2018-11-11

**Authors:** Onur Ozturk, Sanem Arikan, Ayfer Atalay, Erol O. Atalay

**Affiliations:** ^1^ Department of Biophysics Inonu University School of Medicine Malatya Turkey; ^2^ Department of Biophysics Pamukkale University School of Medicine Denizli Turkey

**Keywords:** haplotype, Hb D‐Los Angeles, phylogenetic network analysis, time estimate, β‐globin gene cluster

## Abstract

**Background:**

β‐Globin gene cluster haplotypes associated with the Hb D‐Los Angeles mutation have been reported in many different locations in different populations including Italy, Iran, Thailand, Belgium, Mexico, Holland, and Turkey. In this study, we have identified genetic relationships and formation periods between the haplotypes reported in the world regarding the Hb D‐Los Angeles.

**Methods:**

We comparatively analyzed the RFLP (restriction fragment length polymorphism) data in Denizli region and world populations using Arlequin 3.5 statistical software program. The data obtained from the Arlequin software were then entered into the Phylogenetic Network software to calculate the age estimates and to discover possible links between the haplotypes.

**Results:**

We observed the frequencies of the β‐globin gene haplotypes for the seven polymorphic restriction sites around the world and calculated the estimated time of haplotypes using Network software on the basis of ancestral haplotypes. We performed the phylogenetic network analysis of the haplotypes linked with Hb D‐Los Angeles mutation by processing the data of frequency and age estimations with Network software.

**Conclusion:**

Our period of time results suggests that HAP1 was formed before modern human migration to Asia and/or independent origin of the Hb D‐Los Angeles mutation from other populations. Considering that the population in Denizli region started the Hb D‐Los Angeles mutation past about 40,000 years ago, it can be said that HAP1, HAP15, and HAP21 belong to the gene pool with an external effect. Our period of time results of HAP6 is compatible with published dating results.

## 1 INTRODUCTION

Hb D‐Los Angeles (HGVS name: *HBB*: NG_000007.3:g.71938G˃C) is an abnormal hemoglobin (Hb) with an amino acid substitution of glutamine for glutamic acid at codon 121 of the beta globin. In Denizli province of Turkey, the most common abnormal hemoglobin variant is Hb D‐Los Angeles [β121(GH4) Glu → Gln] among the total abnormal hemoglobin observed (Atalay et al, 2005). Hb D‐Los Angeles has been reported in many different locations in different populations including Italy, Iran, Thailand, Belgium, Mexico, Holland, and Turkey. Researches from these parts of the world show that β‐globin gene cluster haplotypes reported in association with the Hb D‐Los Angeles cases were [ − + + − + + + ], [ + − − − − + + ], [ − + − − + + + ], [ − − + − + + + ] from Turkey (Atalay et al, 2007; Bahadır et al, 2009; Ozturk et al, 2007); [ − + + − + + + ], [ + − − − − + + ], [ − + + − − + − ], [ + − − − − − + ], [ − + − − + + + ], [ − + − + + + + ] from Iran (Mahdavi, Jalali, Kosaryan, Roshan, & Mahdavi, 2015; Rahimi et al, 2006; Yavarian et al, 2009); [ − + + − + + + ] from Thailand (Fucharoen, Changtrakun, Surapot, Fucharoen, & Sanchaisuriya, 2002); and [ + − − − − + + ], [ − − − − − + + ] from India (Patel, Mahson, Patel, Dash, & Das, 2010); [ + − − − − + + ] from Holland, Belgium, Italia, and Mexico (Fioretti et al, 1993; Perea et al, 1999; Yavarian et al, 2009).

There are two different hypotheses regarding the origin of the Hb D‐Los Angeles mutation; firstly, the mutation may have arisen in the Mediterranean area, independent from other populations, and secondly, the mutation may have arisen only once, most likely in Asia, depending on the prevalence in the Punjab (India) and Northwest China (Fioretti et al, 1993). Ozturk Arikan Atalay and Atalay (2016) research results support the hypothesis that the origin of the Hb D‐Los Angeles mutation may have been in the Mediterranean area, independent from other populations rather than from recent Asiatic migrations. The parameter *τ* (tau), calculated from the mismatch distribution, is used to estimate the time since the expansion (*t*) using the equation *t* = *τ* /2*u*, where *u* is the mutation rate per sequence per generation. According to the results based on estimated values of *τ*, the average time since the demographic expansion for normal and Hb D‐Los Angeles populations in Denizli, Turkey, ranged from approximately 42,000 ybp (95% CI; 25,000–58,000) and 38,000 ybp (95% CI; 18,500–62,000), respectively (Ozturk et al., 2016).

We think that studies on genetic relationships of β‐globin gene cluster haplotypes associated with the Hb D‐Los Angeles mutation are important in terms of understanding the mechanisms of mutation formation and having knowledge about the evolution of modern humans (HS, Homo sapiens sapiens). With this point of view, in this study, we have identified genetic relationships and formation periods between the haplotypes reported in the world regarding the Hb D‐Los Angeles. The analysis was performed statistically with Arlequin (ver. 3.5) and Network 5.0.0.3. software depending on the molecular genetic data based on the beta globin gene cluster haplotypes (Bandelt, Forster, & Röhl, 1999; Excoffier, Laval, & Schneider, 2005; Forster, Harding, Torroni, & Bandelt, 1996; Phylogenetic Network Software, https://www.fluxus-engineering.com/sharenet.htm).

## 2 MATERIALS AND METHODS

### 2.1 Haplotype identification and statistical analysis

In our study, beta globin gene cluster haplotypes are the basis of our calculations for our province (Denizli, Turkey) and also for other published results. These haplotype data were already obtained by the polymerase chain reaction–restriction fragment length polymorphism (PCR‐RFLP) procedure on seven polymorphic restriction sites (HincII 5′ to ε, HindIII 5′ to Gγ, HindIII in the IVS‐II 5′ to Aγ, HincII in ψβ, HincII 3′ to ψβ, AvaII in β, and HinfI 3′ to β) in the β‐globin gene cluster as previously reported (Ozturk et al., 2016). We comparatively analyzed the RFLP data in Denizli region using Arlequin 3.5 statistical software program (Excoffier et al., 2005; Falchi et al, 2005). As a result, we identified haplotypes in 18 different frequencies associated with Hb D‐Los Angeles mutation in the region. Similarly, we performed statistical analysis of world populations and applied the test results in Arlequin 3.5 software program with unknown gametic phase such as haplotype analysis and determined the frequencies of haplotypes. The data obtained from the Arlequin software were then entered into the Phylogenetic Network Software to calculate the age estimates and to discover possible links between the haplotypes (Bandelt et al., 1999; Excoffier et al., 2005; Forster et al., 1996; Phylogenetic Network Software, https://www.fluxus-engineering.com/sharenet.htm). The *ρ* (rho) value in Network software measures the age of an ancestral node in mutational units. The mutation age is then converted into years as the result of multiplication by the mutation rate (1 mutation per 20,180 years is the mutation rate for human mtDNA in the stretch np 16,090–16,365). A straightforward way to estimate the age of a particular “root” haplotype, given the mutation rate, is to consider all available descendant individual sequences and take the arithmetic mean over all distances to the root haplotype. This method is referred to as “rho” estimation and can be performed using the freely available software package Network (Morral et al, 1994; Saillard, Forster, Lynnerup, Bandelt, & Nørby, 2000).

## 3 RESULTS

We presented the frequencies of the β‐globin gene haplotypes for the seven polymorphic restriction sites around the world (Table 1). Table 1 summarizes the possible types and frequencies of each haplotype, which were performed by Arlequin 3.5 software program.

**Table 1:** The haplotypes and frequencies of world populations linked with Hb D‐Los Angeles

			Frequencies of world populations							
			Turkey	Iran	India	Thailand	Italia	Holland	Belgium	Mexico
Haplotype	HAP1	− + + − + + +	0.0652	0.0449	0	1	0	0	0	0
	HAP2	+ + + − + + +	0.174	0	0	0	0	0	0	0
	HAP3	− − − − − + −	0.0435	0	0	0	0	0	0	0
	HAP4	− − − − − + +	0.0652	0	0.24	0	0	0	0	0
	HAP5	+ + − − − + +	0.0217	0	0	0	0	0	0	0
	HAP6	+ − − − − + +	0.217	0.584	0.76	0	1	1	1	1
	HAP7	− + + + + + +	0.0435	0	0	0	0	0	0	0
	HAP8	− + − − + − +	0.0217	0	0	0	0	0	0	0
	HAP9	+ + − + + + +	0.0217	0	0	0	0	0	0	0
	HAP10	− − − − − − −	0.0217	0	0	0	0	0	0	0
	HAP11	− + + − − + +	0.0217	0	0	0	0	0	0	0
	HAP12	+ − − − − − +	0.0435	0.18	0	0	0	0	0	0
	HAP13	− − − − − − +	0.109	0	0	0	0	0	0	0
	HAP14	− + − − − − +	0.0435	0	0	0	0	0	0	0
	HAP15	− + − − + + +	0.0217	0.101	0	0	0	0	0	0
	HAP16	+ − − − − + −	0.0217	0	0	0	0	0	0	0
	HAP17	− + + − − − +	0.0217	0	0	0	0	0	0	0
	HAP18	+ − − − + + +	0.0123	0	0	0	0	0	0	0
	HAP19	− + − + + + +	0	0.0449	0	0	0	0	0	0
	HAP20	− + + − − + −	0	0.0449	0	0	0	0	0	0
	HAP21	− − + − + + +	0.0094	0	0	0	0	0	0	0

We calculated the estimated time of haplotypes using Network software on the basis of ancestral haplotype (Table 2). Long, Chakravarti, Boehm, Antonarakis, and Kazazian (1990) inferred that haplotype ( − − − − − − − )was the ancestral human haplotype. Table 2 shows the ancestral haplotype as HAP10. The standard deviations (*SD*) in Table 2 are calculated for both years and sigma mutation unit values. In this analysis, the rho estimation is independent from the demographic parameters but depends on the connections between the haplotypes.

**Table 2:** The age estimation of haplotypes linked with Hb D‐Los Angeles

			Estimated age on the basis of ancestral haplotype			
			Years	Years *SD*	Rho statistic	Sigma *SD*
Haplotype	HAP1	− + + − + + +	94,964.7	18,992.9	4.705	0.94
	HAP2	+ + + − + + +	107,626.6	17,937.7	5.333	0.88
	HAP3	− − − − − + −	13,453.3	1,245.4	0.666	0.66
	HAP4	− − − − − + +	36,324	1,816.2	1.8	0.9
	HAP5	+ + − − − + +	40,360	10,090	2	0.5
	HAP6	+ − − − − + +	59,946.4	19,982.1	2.97	0.99
	HAP7	− + + + + + +	80,720	13,453.3	4	0.66
	HAP8	− + − − + − +	30,270	10,090	1.5	0.5
	HAP9	+ + − + + + +	60,540	10,090	3	0.5
	HAP10	− − − − − − −	Ancestral haplotype			
	HAP11	− + + − − + +	40,360	10,090	2	0.5
	HAP12	+ − − − − − +	38,235.7	19,117.8	1.894	0.94
	HAP13	− − − − − − +	16,816.6	16,816.6	0.833	0.83
	HAP14	− + − − − − +	26,906.6	13,453.3	1.33	0.66
	HAP15	− + − − + + +	73,381.8	18,345.4	3.63	0.90
	HAP16	+ − − − − + −	20,180	10,090	1	0.5
	HAP17	− + + − − − +	30,270	10,090	1.5	0.5
	HAP18	+ − − − + + +	40,360	10,090	2	0.5
	HAP19	− + − + + + +	80,720	16,144	4	0.8
	HAP20	− + + − − + −	80,720	16,144	4	0.8
	HAP21	− − + − + + +	60,540	10,090	3	0.5

Note

Years: refers to the time since the formation of an ancestral haplotype.

**Figure 1:**
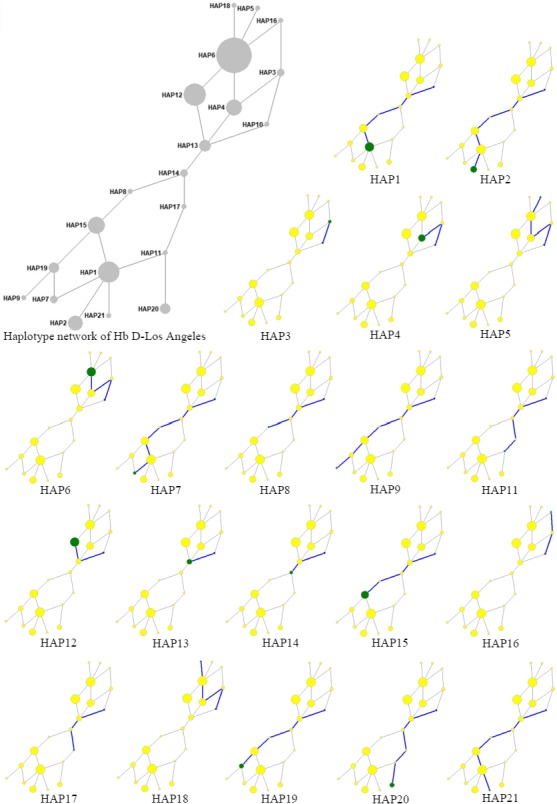
Phylogenetic network analysis of the haplotypes linked with Hb D‐Los Angeles mutation (The gray and yellow circles, which have the same locations in the network, represent the same haplotype numbers. The diameters of the circles are directly proportional to the frequency of the related haplotype. The blue line shows the most probable network connection for the formation of the target haplotype (green circles) from the ancestral haplotype [HAP10])

We performed the phylogenetic network analysis of the haplotypes linked with Hb D‐Los Angeles mutation by processing the data of frequency and age estimations with Network software (Figure 1). This figure shows the phylogenetic analysis of each haplotype, calculated based on the relationship between the loci and time estimates.

## 4 DISCUSSION

Research on network between β‐globin gene cluster haplotypes associated with Hb D‐Los Angeles mutation provides informative insights into population interactions and gene exchange, such as population movements, migrations, and environmental effects on mutation mechanisms. Our study aimed to describe the links between all the reported haplotypes worldwide associated with the mutation based on the possible haplotype tree and time of occurrence.

Ozturk Arikan Atalay and Atalay (2016) inferred that the average time since the demographic expansion for normal and Hb D‐Los Angeles populations ranged from approximately 42,000 (95% CI; 25,000–58,000) ybp (years before present) to 38,000 (95% CI; 18,500–62,000) ybp, respectively, in Denizli, Turkey. These results support the hypothesis that the origin of the Hb D‐Los Angeles mutation may have been in the Mediterranean area, independent from other populations rather than from recent Asiatic migrations in Denizli, Turkey. According to the published data, Homo sapiens neanderthalensis (HN) constitute a group of hominids whose particular morphology developed in Europe during the last 350,000 years under the effect of selection and genetic drift, reaching its final form approximately 130,000 ybp (Klein, 2003). This subgroup of hominids populated Europe and western Asia until the arrival of the first modern humans, Homo sapiens sapiens (HS), approximately 45,000 ybp (Mellars, 1992). Available data on European mtDNA diversity indeed support this view, since most European populations do present a signal of Paleolithic demographic expansion from a small population, which could be dated to about 40,000 ybp (Excoffier & Schneider, 1999). From 50,000 to 46,000 ybp, Homo sapiens entered Europe. Most Europeans today can trace their ancestry to mtDNA lines that appeared between 50,000 and 13,000 ybp (Oppenheimer, 2012).

According to our results, the formation time of the HAP6 [ + − − − − + + ] (also known as the Mediterranean haplotype) approximately ranged from 59,946.4 ± 19,982.1 after the formation of an ancestral haplotype (Table 2). Most of the cases the Hb D‐Los Angeles mutation observed in the world is the highest frequency associated with the HAP6 (Mediterranean [ + − − − − + + ]). Ozturk Arikan Atalay and Atalay (2016) research shows that the Hb D‐Los Angeles mutation developed in the genetic pool of the normal population in Denizli, Turkey, ranged from approximately 38,000 (95% CI; 18,500–62,000) ybp. Our period of time results of HAP6 is compatible with Ozturk Arikan Atalay and Atalay (2016) and Oppenheimer (2012) dating results. When the ancestral haplotype (HAP10) is taken into consideration, the formation of HAP6 is followed by HAP3 and HAP4 networks (Figure 1). Reported from Turkey, Iran, and Thailand, HAP1 [ − + + − + + + ] approximately ranged from 94,964.7 ± 18,992.9 years after the formation of an ancestral haplotype (Table 2). This period of time results suggests that HAP1 was formed before modern human migration to Asia and/or independent origin of the Hb D‐Los Angeles mutation from other populations. HAP1 is followed by HAP13, HAP14, HAP8, and HAP15 networks (Figure 1). Reported from Iranian, Indian, and English populations, HAP19 [ − + − + + + + ] approximately ranged from 80,720 ± 16,144 years after the formation of an ancestral haplotype (Table 2). HAP4 [ − − − − − + + ], which was reported in India and observed with the HAP3 network, occurred approximately 36,324 ± 1,816.2 years after the ancestral haplotype. Reported Iran and Turkey, HAP15 [ − + − − + + + ] was calculated to occur approximately 73,381.8 ± 18,345.4 years after the ancestral haplotype with HAP13, HAP14, and HAP8 connection formation (Table 2, Figure 1). Considering the dating results of Ozturk Arikan Atalay and Atalay (2016), it is understood that HAP15 may be related to the Hb D‐Los Angeles mutation that occurs in a different origin. HAP12 [ + − − − − − + ] and HAP20 [ − + + − − + − ] reported from Iran 38,235.7 ± 19,117.8 and 80,720 ± 16,144 years after the ancestral haplotype, respectively. Finally, reported from Turkey HAP21 [ − − + − + + + ] occurred approximately 60,540 ± 10,090 years after the ancestral haplotype. These dates suggest that HAP 21 is associated with Hb D‐Los Angeles mutation, which appears on the local population in Denizli, Turkey. Considering that the population in Denizli region started the Hb D‐Los Angeles Mutation past about 40,000 years ago, it can be said that HAP1, HAP15, and HAP21 belong to the gene pool with an external effect.

We think that the haplotype network data presented in our work provide useful information to discuss about the evolutionary development and historical migration routes of modern human being. On the other hand, gene exchange could also be the cause of the mutation development, considering the recent publication on the gene exchange in between extinct hominine groups of Denisovans and Neanderthals (Slon et al, 2018). Higher levels of Neanderthal ancestry have been observed in East Asians compared to Europeans. Recent work has proposed that this difference resulted from a dilution of Neanderthal ancestry in Europeans after admixture with an unsampled modern human population (“basal Eurasians”) that had little or no Neanderthal admixture. Others have instead suggested that the higher Neanderthal ancestry observed in East Asians is a result of additional waves of Neanderthal admixture. Analysis of an ancient European genome has shown that at least one additional pulse of Neanderthal admixture occurred in Europe, although this modern human population does not seem to have left present‐day descendants. Additionally, a recent study suggests that part of the Denisovan‐like ancestry found in present‐day East Asians is due to an archaic group more closely related to the sequenced Denisovan genome than the Denisovan‐like ancestry in South Asians and Oceanians, providing support for a two‐pulse model for Denisovan‐like admixture (Dannemann & Racimo, 2018). Considering this information, it is possible to explain the difference between the formation periods of Thailand HAP1 [ − + + − + + + ] and Mediterranean HAP6 [ + − − − − + + ] type haplotypes in our study. The evaluation of such data may contribute valuable information to anthropological, paleoclimatic, archeological, and phylogeographical approaches to human biology throughout the historical periods of time.

## CONFLICT OF INTERESTS

Authors Ozturk, Arikan, Atalay, and O. Atalay declare that they have no conflict of interests.

## AUTHOR CONTRIBUTIONS

SA and AA contributed to data interpretation and manuscript preparation. EOA supervised the study, and provided support in the data interpretation and manuscript preparation.
